# Multi-Omics Studies Unveil Extraciliary Functions of BBS10 and Show Metabolic Aberrations Underlying Renal Disease in Bardet–Biedl Syndrome

**DOI:** 10.3390/ijms23169420

**Published:** 2022-08-20

**Authors:** Emanuela Marchese, Marianna Caterino, Roberta Fedele, Francesca Pirozzi, Armando Cevenini, Neha Gupta, Diego Ingrosso, Alessandra Perna, Giovambattista Capasso, Margherita Ruoppolo, Miriam Zacchia

**Affiliations:** 1 Department of Mental, Physical Health and Preventive Medicine, University of Campania L.Vanvitelli, 80138 Naples, Italy; 2 Department of Molecular Medicine and Medical Biotechnology, University of Naples Federico II, 80131 Naples, Italy; 3 CEINGE—Biotecnologie Avanzate Franco Salvatore, 80145 Naples, Italy; 4 Department of Translational Medical Sciences, University of Campania L.Vanvitelli, 80131 Naples, Italy; 5 Biogem S.c.ar.l., 83031 Ariano Irpino, AV, Italy; 6 Department of Precision Medicine, University of Campania “Luigi Vanvitelli”, 80138 Naples, Italy

**Keywords:** Bardet–Biedl syndrome, urine metabolomics kidney dysfunction

## Abstract

Bardet–Biedl syndrome (BBS) is a rare autosomal recessive ciliopathy resulting in multiple organ dysfunctions, including chronic kidney disease (CKD). Despite the recent progress in the ’ciliopathy’ field, there is still little information on the mechanisms underlying renal disease. To elucidate these pathomechanisms, we conducted a translational study, including (i) the characterization of the urine metabolomic pattern of BBS patients and controls in a pilot and confirmation study and (ii) the proteomic analysis of the BBS10 interactome, one of the major mutated BBS genes in patients, in a renal-epithelial-derived cell culture model. The urine metabolomic fingerprinting of BBS patients differed from controls in both pilot and confirmation studies, demonstrating an increased urinary excretion of several monocarboxylates, including lactic acid (LA), at both early and late CKD stages. Increased urine LA was detected in the absence of both increased plasmatic LA levels and generalized proximal tubular dysfunction, suggesting a possible renal-specific defective handling. The inner medulla renal epithelial (IMCD3) cell line, where *Bbs10* was stably invalidated, displayed an increased proliferative rate, increased ATP production, and an up-regulation of aerobic glycolysis. A mass spectrometry-based analysis detected several putative BBS10 interactors in vitro, indicating a potential role of BBS10 in several biological processes, including renal metabolism, RNA processing, and cell proliferation. The present study suggests that the urine metabolomic pattern of BBS patients may reflect intra-renal metabolic aberrations. The analysis of BBS10 interactors unveils possible novel functions, including cell metabolism.

## 1. Introduction

‘Ciliopathies’ is the definition of a group of rare inherited disorders characterized by high genotypic and phenotypic overlapping, including a number of autosomal recessive syndromic conditions such as Bardet–Biedl (BBS), Joubert (JS), Alstrom (AS), Meckel–Gruber (MKS), Senior–Locken (SLS) and Oral-Facial-Digital type 1 (OFD-1) syndromes. Autosomal dominant disorders include the most common inherited kidney cystic disorder, ADPKD, due mainly to *PKD1-2* mutations [1]. Structural and functional renal abnormalities are cardinal clinical features of most ciliopathies [1]. In fact, chronic kidney disease (CKD) is one of the most common causes of mortality [2,3]. Clinical features of BBS patients combine retinal, renal, and several metabolic abnormalities [4]. According to the literature, the spectrum of renal disorders is quite large. Renal cysts, renal hypo-dysplasia, pelvic dilations, vesico-ureteral reflux, and other urological anomalies have been described in several case reports [4,5]. In one of the largest cohort studies, 6% of BBS patients have been shown to reach end-stage renal disease during childhood [4]; the others may face several situations, including a progressive decline of renal function, as suggested by our recent study [6].

Metabolomics has been applied to the Nephrology field in several areas of research, including ciliopathies [2,7,8]. Metabolomic profiling of the extracellular medium of Pkd1-deficient cells showed reduced glucose and increased lactate concentration, suggesting an increased aerobic glycolysis. Accordingly, kidney-specific Pkd1 inactivation in mice resulted in increased ^13^C-glucose uptake and ^13^C-lactate production on NMR spectroscopy, confirming abnormal glycolytic activation in vivo as well [9]. Subsequently, Podrini et al. demonstrated a broad metabolic reprogramming in ADPKD, including enhanced glycolysis, reduced Krebs cycle and fatty acid oxidation, and enhanced fatty acid synthesis [10]. Similar results were observed in an experimental model of Nephronophthisis, namely jck mice [11], where deregulated peroxisome activity and oxidative stress have been postulated to contribute to cyst progression.

Here, we apply a mass spectrometry-based metabolomic approach to characterize the urine metabolomic fingerprinting of a population of adult BBS patients. A pilot study, including only BBS patients with normal estimated glomerular filtration rate (eGFR), was followed by a confirmation study, in which BBS patients with any eGFR were compared with healthy volunteers and with CKD patients due to other causes. Additional in vitro studies have analyzed the role of BBS10 in renal epithelial physiology, suggesting novel biological roles, such as the involvement in cellular metabolism and RNA processing.

## 2. Results

### 2.1. Pilot Study

**Cohort I.** A cohort of fourteen BBS patients with preserved renal functionality (estimated glomerular filtration rate (eGFR) >60 mL/min/m^2^) and twenty age-gender matched healthy controls were selected. The main features of the patients and controls are represented in Table 1. Briefly, the BBS cohort consisted of seven male and seven female individuals aged 18–43 years old; the mean eGFR and body mass index (BMI) were 99.4 ± 24.5 mL/min/1.73 m^2^ and 30.8 ± 6.5 kg/m^2^, respectively. Additionally, the mean systolic blood pressure (SBP) and diastolic blood pressure (DBP) were 111.3 ± 11.9 mmHg and 80.1 ± 7.3 mmHg, respectively. Retinal dystrophy was present in 13/14 individuals; polydactyly in 10/14 subjects. Controls were matched with patients for age, gender, eGFR, and BMI.

**Urine metabolic profile of BBS patients compared with controls: increased urine lactic acid (LA) excretion was among the most significant alterations**. In total, 21 metabolites discriminated the urine composition of BBS patients compared with controls, as listed in Table 2. Interestingly, lactic acid (LA) was among the most significant metabolites and showed increased urine excretion in BBS patients compared with controls. Moreover, beta-lactic acid, 3-hydroxy-isobutyric acid, and pyruvic acid were over-excreted in BBS patients compared with controls. These molecules are monocarboxylates that are reabsorbed along the proximal tubule (PT) with the same transport mechanism.

**The analysis of Proximal Tubule (PT) functions revealed the absence of generalized tubular dysfunction**. As proximal tubule (PT) reabsorption is considered the main determinant of urine LA excretion, we addressed whether generalized PT dysfunctions characterized BBS patients. Plasma electrolytes, phosphate, and uric acid levels were in the normal range and did not differ between patients and controls (Table 3). No patient showed metabolic acidosis according to blood gas analysis. Glycosuria, another hallmark of PT dysfunction, was absent in all patients studied. No differences were detected in urinary amino acid excretion between patients and controls (Table 3), with the exception of glutamic acid which was almost undetectable in BBS patients and showed a quite low abundance in controls as well. These findings were contrary to the hypothesis of a generalized PT dysfunction in BBS patients, at least at basal and in the absence of stress conditions.

### 2.2. Confirmation Study

**Cohort II.** A total of 30 BBS patients and 32 controls underwent untargeted urine metabolomics analysis. Features of the patients and controls are listed in Table 4. Patients were divided into two groups based on the eGFR. Specifically, patients having an eGFR higher than 90 mL/min/1.73 m^2^ were referred to as BBS_noCKD. Conversely, patients having an eGFR lower than 90 mL/min/1.73 m^2^ were therefore referred to as BBS_CKD. Control subjects included healthy volunteers (ctr_hv) and subjects affected by CKD (ctr_CKD) due to other causes, mainly glomerulonephritis and congenital anomalies of the kidney and urinary tract malformations (CAKUT). The groups of BBS and controls were matched for age, gender, eGFR, and BMI.

**Figure 1:**
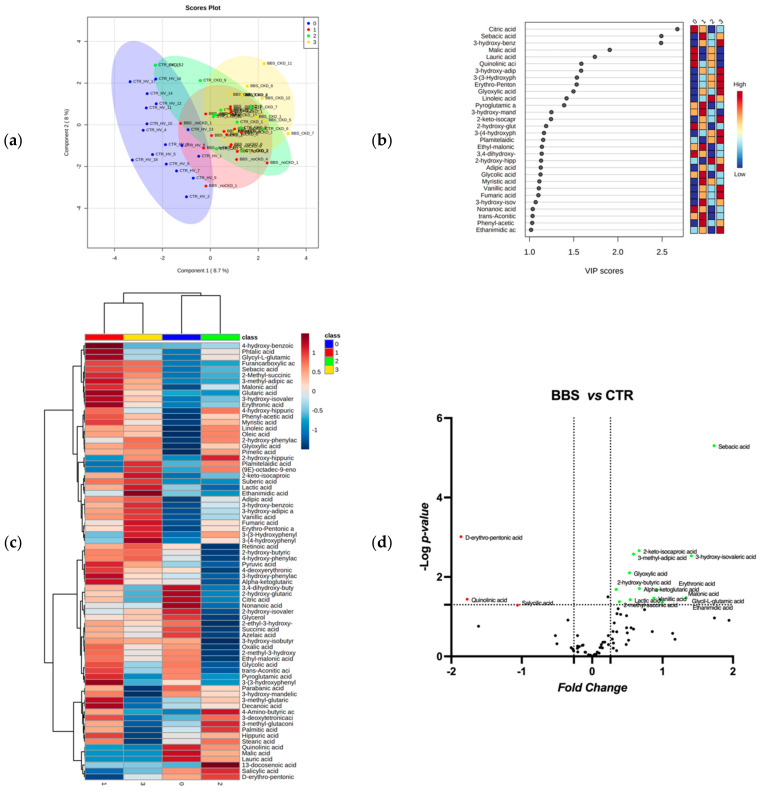
Descriptive discriminant analysis of the urine metabolome of BBS patients. (**a**) The supervised partial least squares-discriminant analysis (PLS-DA) shows the segregation in four-condition analyzed metabolomes, CTR_HV, BBS_noCKD, CTR_CKD, and BBS_CKD, according to component 1, 8.7%, and component 2, 8.0%. (**b**) Discriminant features were identified according to the Variable Importance in Projection (VIP) score. The 30 most important molecules with VIP score values >1.0 are reported. The intensity of the colored boxes represents the relative abundance in each group. The concentrations of the identified metabolites were normalized, log (10) transformed, and Pareto scaled. (**c**) Heatmaps of the average urine metabolite concentrations (μM) in each group (0 = CTR_HV, 1 = BBS_noCKD, 2 = CTR_CKD, and 3 = BBS_CKD). The hits were ranked by *ANOVA*. (**d**) Volcano plot analysis of metabolites significantly different in BBS patients versus controls (BBS, including BBS_noCKD and BBS_CKD, versus CTR, including CTR_HV and CTR_CKD). The relative abundance of each metabolite was plotted against its statistical significance, respectively, reported as Fold Change (log2 fold change) and -log10 (*p*-value). Red and green dots indicate features that presented a *p*-value < 0.05 and a Fold Change < −1.3 or >1.3, respectively. Black dots refer to all the metabolites identified in the dataset whose relative abundance is not significantly different between BBS patients and healthy controls.

**Increased urine LA excretion was observed in all BBS patients, with and without eGFR decline.** An untargeted metabolic approach was used to characterize the BBS patient’s urine metabolome. The GC-MS-based analytical platform was used and about 80 metabolites were correctly identified and quantified. A comprehensive list of the measured metabolites, including metabolite names and their raw concentrations in each patient, is shown in Supplemental Table S1. Univariate and multivariate statistical analyses were employed for selecting the most significant metabolite alterations in urine levels between patients with respect to the relative control group. Specifically, to define the BBS metabolome signature, the datasets were processed according to a supervised partial least squares-discriminant analysis (PLS-DA). Results showed discrete metabolome clustering in four different groups, CTR_HV, BBS_noCKD, CTR_CKD, and BBS_CKD according to the variance of the component 1 = 8.7% and component 2 = 8% (Figure 1a). The Variable Importance in Projection (VIP) measure was used to identify the most discriminant hits between CTR_HV, BBS_noCKD, CTR_CKD, and BBS_CKD (Figure 1b). In particular, the levels of metabolites such as sebacic acid, 3-hydroxy benzoic acid, malic acid, citric acid, lauric acid, and 3-hydroxy adipic acid were able to discriminate (VIP > 1.5) between the analyzed groups, highlighting metabolic abnormalities likely associated with the BBS phenotype. Interestingly, the hierarchical clustering of identified and quantified metabolites shown in the heatmap (Figure 1c) exhibits a distinct pattern in metabolite abundance between the four groups, CTR_HV, BBS_noCKD, CTR_CKD, and BBS_CKD. In detail, the dendrogram indicates a clear clusterization in metabolite abundance profiles of two BBS groups (BBS_noCKD and BBS_CKD), characterized by a reasonable difference in urine metabolite levels with respect to controls. In order to highlight the molecule alteration more related to BBS, a binary comparison was performed according to the volcano plot, and all the metabolites showed significant differences between the two groups (BBS, including BBS_noCKD and BBS_CKD, versus CTR, including CTR_HV and CTR_CKD) are shown in Figure 1d.

**Figure 2:**
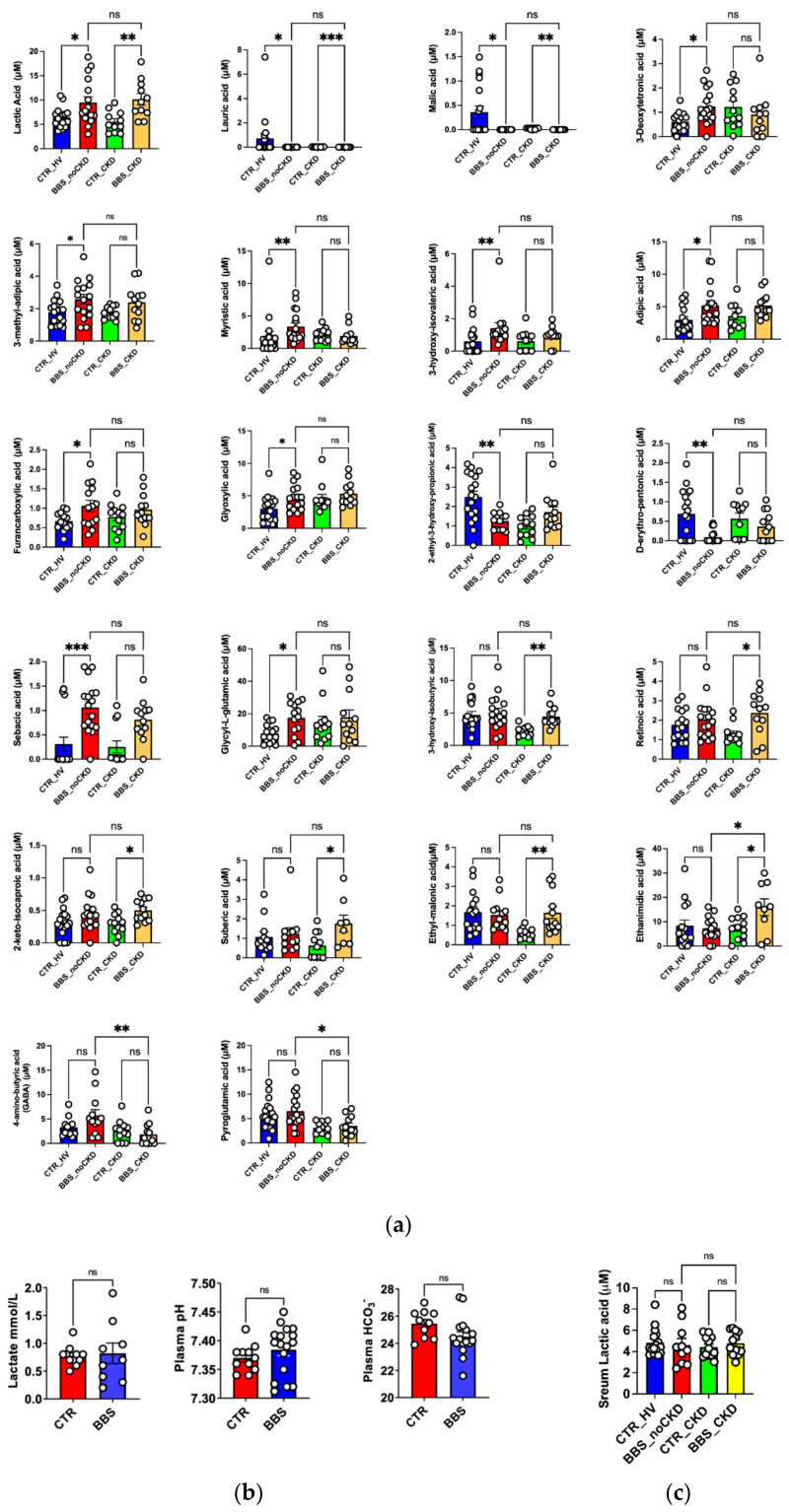
The differential urine metabolomic profiling in distinct cohorts of BBS patients. (**a**) Significantly differential urine metabolite concentrations in considered groups, CTR_HV (*n* = 18), BBS_noCKD (*n* = 18), CTR_CKD (*n* = 13), and BBS_CKD (*n* = 13). (**b**) Serum pH, bicarbonate, and lactic acid levels showed no difference between patients and controls. The analysis demonstrated that the total BBS population did not display a significant variation in serum lactic acid levels compared to the control population. (**c**) Serum LA levels did not display significant variation when the total BBS population was compared with healthy subjects and CTR_CKD, respectively. The serum LA was measured by LC/MSMS. Plots represent the concentrations of the analytes (means ± SEM). In binary comparison, the significant difference was evaluated by performing a parametric t-test with Welch correction in normally distributed datasets or the Mann–Whitney test in not-normally distributed datasets. In multiple comparisons, the significant differences between groups were evaluated by performing an ordinary one-way ANOVA test and Hold–Sidak’s multiple comparison test in normally distributed datasets or the Kruskal–Wallis test and Dunn’s multiple comparison test in not-normally distributed datasets. The normal distribution was verified according to the D’Agostino and Pearson test. (* *p* < 0.05, ** *p* < 0.01, *** *p* < 0.001, ns = not significant).

Moreover, as in the pilot study, the relative levels of lactic acid in BBS groups (BBS_noCKD and BBS_CKD) were confirmed as more abundant with respect to controls. To detect additional metabolites, showing significant differences in the BBS conditions compared to controls, a binary comparison was performed according to the volcano plot. The volcano plot analysis revealed that 14/80 (17.5%) metabolites were significantly increased, and 3/80 (3.7%) metabolites were significantly reduced in urine from BBS patients in comparison with the CTRL. Specifically, the binary comparison revealed specific metabolites, 2-keto-isocaproic acid, 3 methyl adipic acid, 3-hydroxy-isovaleric acid, Glyoxylic acid, alpha-ketoglutaric acid, 2-hydroxy-butyric acid, Erythronic acid, Glycil L-glutamic acid, Vanillic acid, Lactic acid, Malonic acid, 2-Methylsuccinic acid, and Ethanimidic acid as more significantly (*p* < 0.05) abundant in the BBS group; conversely, D-erythro pentonic acid, Quinolinic acid, and Salicylic acid results were less significantly (*p* < 0.05) abundant in BBS group. Finally, to define the details of the pathological metabolomic signature of BBS disease, a univariate analysis was carried out. The normal distribution of the urine metabolite concentrations was verified and significant differential profiles were observed evaluating the metabolite concentrations in distinct groups, CTR_HV, BBS_noCKD, CTR_CKD, and BBS_CKD. Specifically, the lactic acid, lauric acid, and malic acid results were significantly altered in BBS_noCKD with respect to CTR_HV as well as in BBS_CKD and CTR_CKD, thus suggesting specific metabolite dysregulation as the major signature of the urine metabolome of BBS patients (Figure 2a). Metabolites such as 3-deoxytetronic acid, 3-methyl-adipic acid, myristic acid, 3-hydroxy-isovaleric acid, adipic acid, furancarboxylic acid, glyoxylic acid, 2-ethyl-3-hydroxy-propionic acid, D-erythro-pentonic acid, sebacic acid, and Glycyl-L-glutamic acid results were significantly altered in BBS_noCKD with respect to CTR_HV, revealing a metabolomic signature independent with respect to the decline of renal function (Figure 2a). Conversely, metabolites such as 3-hydroxy-isobutyrric acid, retinoic acid, 2-keto-isocaproic acid, suberic acid, and ethyl-malonic acid results were specifically altered when the BBS_CKD was compared to CTR_CKD, highlighting the key role of BBS in the progressive onset of chronic kidney disease (Figure 2a). Only a few metabolites, such as ethanimidic acid, 4-amino-butyrric acid, and pyroglutamic acid results were chronic kidney disease dependent as their results were altered when BBS_noCKD was compared to BBS_CKD (Figure 2a).

**Increased urinary LA excretion did not correlate with increased serum LA levels and with acid-base imbalance.** LA is known to be freely filtered by the glomerulus and reabsorbed along the PT via monocarboxylate transporters [12]. Our data suggest the absence of generalized PT dysfunction. We then addressed whether increased urinary LA resulted from an increased filtration load. To this end, serum LA levels and acid-base status were assessed in patients and controls. All patients showed lactic acid levels in the normal range (lower than 1.5 mmol/L); serum pH and bicarbonate levels showed no difference among patients and controls on the blood gas analyzer (Figure 2b). For a more sensitive measurement, serum LA levels were analyzed with LC-MS/MS; this analysis did not reveal differences between patients and controls, as shown in Figure 2c. These findings suggest that increased urine LA excretion was unlikely to be dependent on the increased filtrating load.

### 2.3. In Vitro Studies

**Figure 3:**
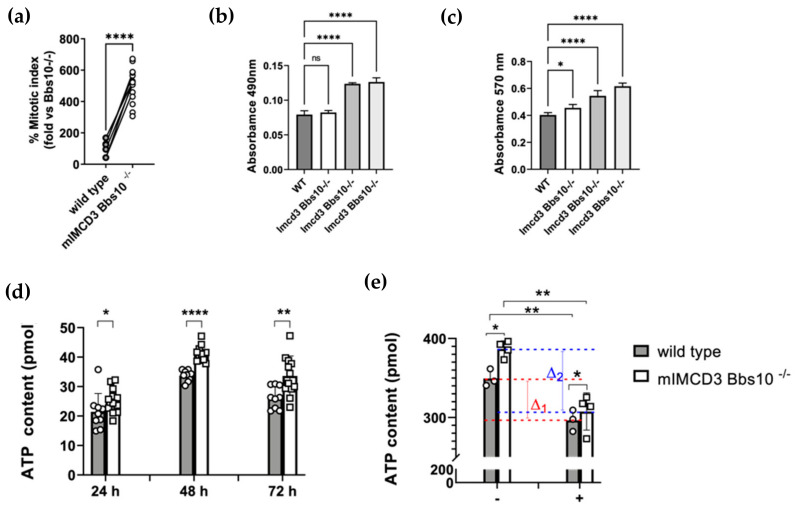
*Bbs10* deficiency is associated with an increase in cellular proliferation rate and enhanced basal ATP production. (**a**) IMCD-*Bbs10*^-/-^ showed an increase in proliferation compared to wild type cells. The mitotic index was calculated as the ratio of the number of cells undergoing mitosis to the total number of cells and expressed as a percentage. (**b**) Absolute quantification of cell survival was performed by measuring the absorbance at λ = 490 nm. The difference between the absorbance of wild type and IMCD3-*Bbs10*^-/-^ cells was highly significant, (**c**) The MTT assay demonstrates significantly increased cell viability in IMCD3-*Bbs10*^-/-^ cells compared to control cells. At a cell density of 0.5 × 10^4^ cells/well, all three knockout cells showed significantly higher absorbance at λ = 570 nm after 24 h of growth. The assay was performed in triplicate and included three different IMCD3-*Bbs10*^-/-^ clones. (**d**) The ATP content in IMCD3-*Bbs10*^-/-^ cells and *wild type cells* at the indicated times after plating. (**e**) Measurement of ATP content in IMCD3-*Bbs10*^-/-^ cells and wild type cells treated in the presence of oligomycin for 5 h. Data are shown as the means ± SEM and are representative of three independent experiments, each performed in triplicate. In binary comparison, the significant difference was evaluated by performing a parametric t-test with a Welch correction in normally distributed datasets or Mann–Whitney test in not-normally distributed datasets. In multiple comparisons, the significant differences between groups were evaluated by performing an ordinary one-way ANOVA test and Hold–Sidak’s multiple comparison test in normally distributed datasets and Kruskal–Wallis test and Dunn’s multiple comparison test in not-normally distributed datasets. The normal distribution was verified according to the D’Agostino and Pearson test. (* *p* < 0.05, ** *p* < 0.01, **** *p* < 0.0001, ns = not significant).

**IMCD3-Bbs10^-/-^ cells showed an increased proliferation rate and increased ATP production.** A stable cell line lacking *Bbs10* was set up in kidney- epithelial cells IMCD3 using CRISPR-Cas9 technology (IMCD3-*Bbs10*^-/-^). We have already shown that IMCD3-*Bbs10*^-/-^ cells showed a deficit of primary cilium formation after starvation according to previous studies [13,14]. Our results demonstrated that IMCD-*Bbs10*^-/-^ cells proliferated at a higher rate than the parental line (Figure 3a) and the elevated mitotic rate was also supported by clonogenic assay (Supplemental Figure S1), cell survival test, and MTT assays (Figure 3b,c). Intriguingly, proliferating IMCD-*Bbs1*0^-/-^ cells exhibited an increase in ATP production of about 17.9%, 22.3%, and 27.8% after 24, 48, and 72, respectively (Figure 3d). After oligomycin treatment, an inhibitor of mitochondrial ATP-synthase, we observed a decreased ATP content in wild type cells compared with cells treated with the vehicle, as expected (Figure 3e). ATP content was reduced after treatment in mIMCD3-*Bbs10*^-/-^ cells as well: the effect of the antibiotic was even higher in mIMCD3-*Bbs10*^-/-^ cells than in wild type cells, suggesting a preserved ability of mitochondrial ATP production (Figure 3e).

**Figure 4:**
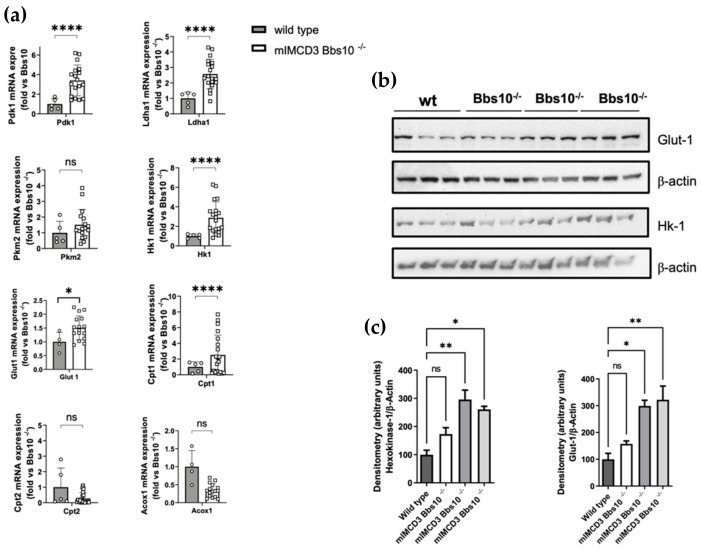
*Bbs10* depletion affects energy metabolism in kidney-derived epithelial cells. (**a**) Relative mRNA expressions levels of key metabolic enzymes including Pdk1, Ldh1, Pkm2, Hk-1, Cpt-1, Cpt2, Acox-1, and Glut-1 (H) in IMCD3-*Bbs10^-/-^*cells (*n* = 20) and wild type cells (*n* = 5). Data are presented as mean ± SEM and are normalized to wild type controls as the *y*-axis is indicated by the fold. The statistical significance of the difference in mRNA expressions between conditions (IMCD3-*Bbs*10^-/-^ cells and wild type controls) was evaluated by an unpaired parametric t-test with Welch correction (* *p* < 0.05, ** *p* < 0.01, **** *p* < 0.0001, ns = not significant). (**b**) Western blot analysis of the expression of hexokinase and Glut-1 in IMCD3-*Bbs10^-/-^* cells and wild type. β-actin was used as the loading control. (**c**) Quantitative densitometric analysis was performed for data analysis and showed an increase in Glu-1 and Hk-1 also at protein levels (*p*-value < 0.05). Data are shown as the means ± SEM and are representative of three independent experiments, each performed in triplicate.

To better elucidate the effect of *Bbs10* depletion on energy metabolism, the analysis of the mRNA levels of the main glycolytic enzymes was performed (Figure 4a). The significant increase in Pyruvate dehydrogenase kinase isozyme 1 (Pdk1, difference: +2.5, *p* < 0.0001), L-lactate dehydrogenase A chain (Ldh1, difference: +1.5, *p* < 0.0001), Hexokinase-1, (Hk1, difference: +2.0, *p* < 0.0001), and Glucose transport-1 (Glut1,difference: +0.5, *p* < 0.05) was observed in mIMCD3 Bbs10^-/-^cells compared to wild type cells, indicating an increased aerobic glycolysis, known as the Warburg effect. The high expression of Glut1 and Hk1 was confirmed at protein levels as well (Figure 4b,c).

**Figure 5:**
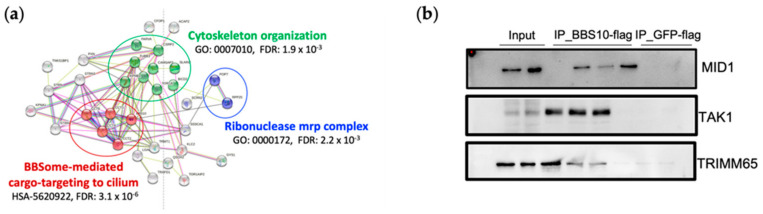
BBS10 protein-protein interaction (PPI) studies. (**a**) The BBS10 putative interactors were enriched for molecular processes in STRING (Search Tool for the Retrieval of Interacting Genes software). Clusters related to Cytoskeleton organization (GO:0007010, FDR 1.9 × 10^−3^), Ribonuclease mrp complex (GO:0000172, FDR 2.2 × 10^−3^), and BBSome-mediated cargo-targeting to cilium (HSA-5620922, FDR 3.1 × 10^−6^) had results that were significantly enriched. (**b**) The more interesting BBS10-flag interactors were validated by Western blot analysis. The immunoprecipitates of the BBS10-flag were loaded on the SDS page. GFP-flag immunoprecipitates were used as a negative control. Each lane represents the immunoprecipitate derived from three different BBS10-flag cell clones. MID1: E3 ubiquitin-protein ligase Midline1; TAK1: also known as mitogen-activated protein 3 kinase 7 (MAP3K7); and TRIM65: Tripartite motif-containing protein.

**BBS10 physically interacts with MID1 and TAK1, known modulators of metabolic pathways, and several extra-ciliary proteins.** To better analyze the role of BBS10 in renal epithelial cell functions, protein-protein interaction (PPI) studies were carried out, aiming at the identification of putative interactors of BBS10 protein, by using a mass spectrometry-based proteomic approach. A HEK293 cell line stably expressing BBS10-flag protein was generated and a cell line expressing the GFP-flag vector was used as a control. In order to isolate and identify specific and a-specific BBS10 protein interactors, two independent immunoprecipitation experiments (IP) were performed by using the BBS10-flag protein and GFP-flag protein, respectively. The protein macrocomplexes were isolated and the protein components were tryptic digested, then analyzed by nano-LC-MS/MS and subsequent quantitative proteomic analysis. The list of total protein components involved in the BBS10 interactome is provided in Supplemental Table S2. The table summarizes the details of the mass spectrometry protein identification of BBS10 putative interactors. The significant BBS10 interactors were selected as the more abundant proteins in the BBS10 interactome with respect to the GFP no-specific interactome, according to the ‘intensity value’ (Table S2) of each identified protein in each biological replicate (*p*-value < 0.05). Finally, a significant protein dataset was processed to avoid a bioinformatics enrichment analysis highlighting the main cellular pathways and processes enriched by the BBS10 interactome. According to the cluster enrichment analysis performed by STRING (Search Tool for the Retrieval of Interacting Genes software), the BBS10 interactors are grouped according to Gene Ontology functional terms in several extra-ciliary functions, including Cytoskeleton organization (GO:0007010, FDR 1.9 × 10^−3^) and Ribonuclease mrp complex (GO:0000172, FDR 2.2 × 10^−3^); furthermore, the reactome pathway annotation showed the formation of a significant cluster as BBSome-mediated cargo-targeting to cilium (HSA-5620922, FDR 3.1 × 10^−6^) (Figure 5a). To test the authenticity of the identified proteins as putative BBS10 interactors, the presence of representative proteins in the eluates from BBS10-flag proteins was confirmed by immunoblotting. The bound proteins were eluted from the resins and analyzed by Western blot using specific antibodies for the three selected interactors (MID1, TAK1, and TRIMM65). Negative controls confirmed the specificity of the interaction between BBS10 and the proteins analyzed.

Two proteins in the dataset could be specifically linked between BBS10 depletion and the Warburg effect, possibly via mTORC1 up-regulation: the ubiquitin E3 ligase, MID1/TRIM18, and TAK1, also known as MAP3k7 (Figure 5b). Moreover, another metabolism-related protein, GYS1, has been found among the significant BBS10 interactors (Table S2).

## 3. Discussion

This study demonstrates significant differences in the urine metabolomic fingerprinting of BBS patients compared with controls, including high urinary excretion of monocarboxylates, such as lactic acid (LA); these data were paralleled by the evidence of increased LA production in an in vitro model of renal epithelial cells lacking one of the major BBS proteins, suggesting a possible role of BBS proteins in regulating renal metabolism.

Multiple metabolomics technologies have allowed the identification of several urine metabolites that are listed in the free available urine metabolome database (UMDB: http://www.urinemetabolome.ca, accessed on 20 May 2022) [15]. Currently, the UMDB contains information on almost 3000 detectable metabolites that have been associated with hundreds of different clinical entities [16]. These molecules are the most hydrophilic metabolites and their abundance in urine is a function of (1) the plasmatic concentration and binding rate to plasmatic proteins; (2) their tubular reabsorption and/or secretion. At present, little information is available in the literature on urine metabolome composition in autosomal recessive syndromic ciliopathies [1]. The present manuscript describes, for the first time, the urine metabolic profile of patients suffering from BBS. Profiling analysis highlights discriminant metabolomes characterizing the four analyzing groups CTR_HV, BBS_noCKD, CTR_CKD, and BBS_CKD, leading to defined specific metabolic abnormalities associated with the BBS phenotype and no specific metabolic abnormalities simply linked to kidney failure. Unsurprisingly, the unbias analysis indicates a clear difference in urine metabolites abundances of BBS groups with respect to controls. Specific metabolite dysregulation (lactic acid, lauric acid, and malic acid) has been found as the major signature of the urine metabolome of BBS patients. Despite this, a larger set of metabolite alterations affects BBS patients independent of the decline of renal function. In addition, observed abnormal levels in specific metabolites, such as 3-hydroxy-isobutyrric acid, retinoic acid, 2-keto-isocaproic acid, suberic acid, and ethyl-malonic acid seem to be to the key role of BBS in the progressive onset of chronic kidney disease. Finally, a few metabolites, such as ethanimidic acid, 4-amino-butyrric acid, and pyroglutamic acid results showed chronic kidney disease dependence.

Increased urinary excretion of LA was among the most significant alterations in both BBS patients with normal and declined renal function, compared with the respective controls. LA is the end product of anaerobic glycolysis. Micropunture experiments demonstrated that LA is freely filtered by the glomerulus and is almost all reabsorbed along the PT, with less than 2% of urine excretion [12]. In normal conditions, even after starvation and glucose deprivation, the cortex produces little amounts of lactate; conversely, the medulla generates lactate from anaerobic glycolysis, which is then recycled and reabsorbed along the PT where it represents the major substrate for gluconeogenesis [17]. Monocarboxylate transporters (MCT) are transmembrane proteins mediating monocarboxylate uptake across the membrane, including hormones, nutrients, and drugs. These transporters are encoded by the large SLC16 family of solute carriers, including 14 known members. Four members (MCT1, 2, 3 and 4) mediate proton-dependent transport of monocarboxylates such as L-lactate, pyruvate, and ketones [18]. Additionally, sodium-dependent lactate reabsorption has been described and is mediated by members of the SLC5 family, namely, SLC5A8 (SMCT1) and SLC5A12 (SMCT2). In contrast to proton-dependent MCTs transporters that have been shown on the basolateral membrane of the nephron, SMCT1 and SMCT2 staining studies demonstrated their presence on the apical membrane of PT cells [19]. In accordance with its reabsorption along the PT, increased urinary LA excretion occurred in a patient with Fanconi syndrome [20]. Thirumurugan et al. showed that urine LA/creatinine excretion was higher in patients with Fanconi syndrome than in patients with no kidney impairment. Interestingly, this study showed a surprisingly high LA excretion in BBS patients and other congenital abnormalities of the kidney (CAKUT), consistent with our data. To address whether increased urine LA abundance in BBS patients was the result of increased filtrating load, we measured the serum levels of LA and investigated the acid-base status in patients and controls, using first morning serum samples after overnight fasting. Our results indicate that neither serum LA levels nor acid-base status differed between patients and controls, suggesting a possible impaired renal handling in BBS patients. However, the present study cannot exclude that BBS patients may undergo an increased LA production at night or increased daily fluctuation; this interpretation requires additional investigation. However, to analyze if increased LA excretion was a marker of defective PT function, we addressed whether additional PT dysfunction was present in our cohort. Glycosuria and aminoaciduria were absent, and no acid-base, hypouricemia, or alterations in plasma phosphate were found, findings that are against a possible Fanconi-like generalized PT dysfunction. Whether BBS patients have specifically defective monocarboxylate reabsorption is another possibility. Little is known about the regulation of monocarboxylate transporters; it has been described that acid-base status and intracellular metabolic status may affect their functions. Touati et al. reported increased urinary LA in patients with respiratory chain disorders, supporting the hypothesis that increased urinary excretion may be the result of impaired energetic sources [21]. MCTs expression has been shown to depend on the concentrations of their substrates and/or signals arising from cellular metabolism. The hypothesis that abnormalities in intracellular metabolism result in LA accumulation in cells, with consequently reduced reabsorption, was tested. LA has been found highly abundant in the cell culture medium of another model of ciliopathy, namely, Pkd1-mutant cells, due to enhanced anerobic glycolysis [22]. Our study indicates a similar effect in inner medulla-derived renal epithelial cells lacking Bbs-10, one of the major BBS genes [23]. IMCD3 Bbs10^-/-^ showed an increased expression of glycolytic enzymes, suggesting a process similar to the Warburg effect. Increased glycolysis may serve to compensate for other defective metabolic processes. Fatty acids (FAs) are known as the preferred substrates for ATP generation in the PT. FA oxidation is the pivotal process of FA catabolism and depends on both peroxisome and mitochondria function [24]. The mechanism underlying increased glycolysis is unknown. The interaction of BBS10 with MID1 and TAK1 was an additional attractive finding in the delineating picture associating BBS with intrarenal metabolic dysfunction. MID1, known also as TRIM18, has been described to bind alpha4 (α4) and its associated phosphatase, PP2A, preventing PP2A from mTORC1 signaling inhibition; thus, MID1 dis-inhibits the mTOR signaling pathway. The interaction with BBS10 could lead to a deregulation of mTOR, with consequent metabolic derangements. Tak1 is a serine/threonine kinase, also known as MAP3K7. It is crucial for normal embryonic development, and the genetic inactivation of its interactor, TAB1, is associated with heart, lung, and kidney defects and resulted in embryonic lethality. In the kidney, TAK1 is a major upstream molecule mediating TGF-β1-induced MKK3 activation, leading eventually to increased collagen chain synthesis in mesangial cells [25]. In addition, kidney fibroblast TGF-β-induced fibronectin expression is mediated by TAK1 through mTORC1 and mTORC2 signaling [26]. Finally, GYS1 has been found among the significant BBS10 interactors. The protein catalyzes the addition of glucose monomers to the growing glycogen molecule and is activated as a response to high intracellular glucose concentrations. The presence of GYS1 in protein macro-complexes, involving BBS10, together with the up-regulation of the glycolytic enzyme rate allows us to reinforce the hypothetic role of BBS 10 in energy metabolism.

This study has some limitations, however. The sample size is relatively small to provide firm conclusions on the urine metabolic composition of BBS patients, and a larger study is required to confirm our results. However, given the low incidence of BBS, the study may serve as a pilot study to be confirmed in a larger cohort population. Moreover, BBS patients enrolled in the study have different genotypes, while we used a single in vitro model to try to explain the molecular basis underlying urine metabolic aberrations. The following reasons prompted us to choose a single cellular model, namely *Bbs10* depletion: (1) the high prevalence of *BBS10* mutations among patients; (2) the strong clinical overlapping among BBS patients with different genotypes; and (3) the evidence that BBS proteins form multiprotein complexes with putative redundant functions.

Finally, the exact mechanisms underlying the up-regulation of aerobic glycolysis in IMCD3-*Bbs10*^-/-^ cells remain to be elucidated and the data showing putative BBS10 interactors suggest possibilities requiring further analysis.

In conclusion, this study provides data suggesting a unique urinary metabolic profile of BBS patients compared with healthy volunteers and patients with CKD due to other causes. Increased urine LA excretion was among the most significant alterations and did not correlate with increased plasma levels as the result of defective renal handling. Reduced PT re-absorption, possibly due to an increased renal cellular production, can contribute to increased LA excretion, as indicated by the up-regulation of glycolytic enzymes in IMCD3-Bbs10^-/-^ cells. Interestingly, these cells showed biological features shared with other ciliopathies, such as an abnormal proliferative rate and an increased ATP production and anaerobic glycolytic pathway, providing additional examples of commonalities among different ciliopathy models.

## 4. Materials and Methods

### 4.1. BBS Cohort

Adult patients with a clinical diagnosis of BBS according to Beales criteria [27] were enrolled in the study. Post-axial polydactyly was defined as the presence of an extra finger, retinal dystrophy was assessed after ocular examination, and obesity was defined as a body max index over 30 Kg/m^2^. An intellectual disability referred to a defect in writing, spelling, speaking, or memories/social/coordination defects (REF). Kidney function was assessed by estimating the glomerular filtration rate (eGFR) using the CKD-Epidemiology Collaboration Equation (CKD-EPI) with a standardized plasma creatinine measurement (REF); arterial blood gas and urinary and plasma electrolytes were measured by standard analysis using the blood gas analyzer (ABL 90 Flex analyzer, RAdiometer, Danmark); and uric acid and phosphate plasma levels were measured as standard care. A control group consisting of individuals with matched age, gender, BMI, and eGFR was enrolled.

### 4.2. Urine Collection and Preparation

From fasting subjects, the second urine of the day was collected in sterile containers and transported on ice to prevent contamination and degradation. Cells and other insoluble debris were removed by centrifugation at 2000 rpm × 10 min. The supernatants were aliquoted to avoid the freeze-thaw cycles that could alter urinary components. For each sample, one aliquot was used to estimate creatinine concentration with the automatic analysis system BM/Hitachi 904 as described [3]. To normalize metabolite differences affected by hydration status, a urine volume containing 0.5 µmol of creatinine was analyzed. The remaining aliquots of urine samples were stored at −80 °C until utilization. A sample volume containing 0.5 µmol of creatinine was brought to pH 14 by adding NaOH 30% (sodium hydroxide) after the mixtures were incubated with 0.5 mL of hydroxylamine hydrochloride at 60 °C for 1 h for oxidation. Subsequently, many drops of H_2_SO_4_ 2,5 M (sulphuric acid) were added to the sample for acidification until pH 1 was reached. Dimethylmalonic acid (10 mg/mL in 1:1 of H_2_O/CH_3_CH_2_OH (v:v)), pentadecanoic acid (10 mg/mL in CH_3_CH_2_OH), and tropic acid (10 mg/mL in H_2_O) (Sigma-Aldrich, St. Louis, MO, USA) were added to each sample as internal standards, with final concentrations of 10 µM [28]. After, samples were subjected to metabolite extraction with 2 mL of ethyl acetate and centrifuged at 3000 rpm for 5 min. For each sample, the extraction was repeated three times. After centrifugation, the organic phases were collected and saturated with approximately 1 g of Na_2_SO_4_ (Sigma-Aldrich, St. Louis, MO, USA) for 60 min at RT to remove water residue. Then, each extract was centrifuged at 3000 rpm for 10 min and evaporated at 40 °C under a gentle nitrogen flow. In the end, the samples were derivatized in 50 µL of N,O-bis (trimethylsilyl)trifluoroacetamide (Sigma-Aldrich, St. Louis, MO, USA) at 60 °C for 30 min. The mixture was cooled at RT and transferred into 250 µL conical glass inserts in order to carry out the subsequent GC-MS analysis.

### 4.3. GC-MS Analysis

GC-MS analysis was performed using an Agilent 7890A (Agilent Technologies, Santa Clara, CA, USA) gas chromatograph coupled with an Agilent 5975C (Agilent Technologies, Santa Clara, CA, USA) mass spectrometer on a GC column HP-5MS; 30 m × 0.250 mm × 0.25 µm. The GC was equipped with a split-mode capillary injection port held at 280 °C with a split ratio of 10:1. The chromatographic gradient was programmed from 70 to 280 °C at a rate of 10 °C/min with a helium flow of 1 mL/min. The mass spectrometer worked in scan mode from 50 to 650 amu scan range, using a 6 min solvent delay. The metabolite identification was performed by the NIST and Wiley mass spectra library (release 2008) and the MSD Productivity ChemStation software (Agilent Technologies, Santa Clara, CA, USA). Using the ChemStation software, each compound was compared with mass spectra present in the NIST spectra library, looking for fragmentation patterns, mass charge ratios, and peak abundance. For each compound a list of compound similarities was obtained; peaks with a similarity index of more than 80% were given a compound name, while those having less than 80% similarity were listed as unknown metabolites. Compounds were quantified using the areas of total ion chromatograms, comparing the area of each compound with the area of the internal standards used at known concentrations. Concentrations of metabolites were expressed as mmol metabolite/mol creatinine. The areas of internal standard, added to the initial mixture, represent the internal quality controls.

### 4.4. Serum Collection and Preparation

Blood samples were obtained between 8:00 and 10:00 a.m. after an overnight fast. Blood was centrifuged (10 min, 2000× *g* at 4°C) and plasma was aliquoted into separate polypropylene tubes that were immediately stored at −80°C. One 10-μL aliquot was analyzed with the Biocrates Absolute IDQ p180 kit (Biocrates Life Science AG, Innsbruck, Austria). The plasma samples were processed as recommended by the manufacturer and analyzed on a Triple Quad™ 5500+ System QTrap-Ready (AB Sciex) coupled with an Agilent 1260 Infinity II HPLC equipped with an Agilent C18 HPLC column [29].

### 4.5. Serum Lactate Measurement

LA was measured by LC-MS/MS, as elsewhere reported [30]. Briefly, a volume of 500 μL of cold methanol was added to each serum sample. The supernatant was collected and dried under nitrogen. The dried supernatant was dissolved in methanol containing labeled standards at a concentration of 10 microM. The analyses were performed using an API 4000 triple quadrupole mass spectrometer (Applied Biosystems-Sciex, Toronto, ON, Canada) coupled with an 1100 series Agilent high-performance liquid chromatograph (Agilent Technologies, Waldbronn, Germany). The MS/MS analysis of LA was performed by using a Multiple Reaction Monitoring (MRM) experiment (Q1 89.00; Q3 59.00) [31].

### 4.6. Statistical Analysis

Univariate statistics were calculated with GraphPad Prism 9.0 and the results are presented as the mean ± standard error of the mean (SEM). The statistical significance of the difference in metabolite samples (urine and serum) concentrations between two different groups was evaluated by parametric (unpaired t-test with Welch correction) or non-parametric (Mann–Whitney test) tests. The significant difference between multiple groups was evaluated by performing an ordinary one-way ANOVA test and Hold–Sidak’s multiple comparison test in normally distributed datasets and Kruskal–Wallis test and Dunn’s multiple comparison test in not-normally distributed datasets. The outlier samples were removed from the metabolome analysis The normal distribution was verified according to the D’Agostino and Pearson test. Multivariate statistical analysis and metabolite set enrichment analysis was performed using MetaboAnalyst 4.0 (http://www.metaboanalyst.ca, accessed on 20 April 2022) [7]. The normalized metabolic dataset was log_2_ -transformed and scaled according to the Pareto scaling method. Dataset homogeneity was evaluated by partial least squares-discriminant (PLS-DA) to acquire an overview of the data and identify potential severe outliers. Partial least squares-discriminant analysis (PLS-DA) was used to maximize the covariance between the independent variables (metabolites) and the corresponding dependent variable Y for predictive feature identification. The variable importance on projection (VIP) was estimated. VIP is a weighted sum of squares of the PLS loadings taking into account the amount of explained variation in each dimension. The Human Metabolome Database was used to assign the correctly numbered ID (HMDB ID) to each differentially abundant metabolite.

### 4.7. Cell Cultures and Treatments

mIMCD3 cells (Murine inner medullary collecting duct cell line, ATCC no. CRL-2123) were cultured with Dulbecco’s Modified Eagle Medium: Nutrient Mixture F-12 (DMEM/F-12) (Gibco, Pakington, UK) supplemented with a 10% fetal bovine serum (EuroClone, Pakington, UK) and 2 mM L-Glutamine (Sigma-Aldrich, St. Louis, MO, USA), at 37 °C in a 5% CO_2_ atmosphere. HEK 293T/17 cells (human embryonic kidney, ATCC no. CRL-11268) were cultured with high glucose in Dulbecco’s Modified Eagle Medium (DMEM) (EuroClone, Pakington, UK) supplemented with a 10% fetal bovine serum (EuroClone, Paington, UK) and 4 mM L-Glutamine (Sigma-Aldrich, St. Louis, MO, USA) at 37 °C in a 5% CO_2_ atmosphere. For the oligomycin treatment, 0.2 × 10^6^ cells/mm^2^ were seeded into the wells of a 24-well microplate (Costar, Corning Inc., Corning, NY, USA). The medium was replaced 48 h after seeding with a fresh medium containing 30 mg/mL of oligomycin A (Sigma-Aldrich, St. Louis, MO, USA).

### 4.8. Generation of a Cell Line Lacking Bbs10 Using CRISPR/Cas9 Technology

For Bbs10 gene knockout by the CRISPR/Cas9 technique, mIMCD3 wild type cells were seeded at a density of 1.5 × 103 cells/mm^2^ in a 10 cm diameter plate and kept in culture in a medium without antibiotics. After 24 h, the cells were transfected with 12 µg of “Bbs10 CRISPR/Cas9 KO Plasmid (h2)” (Santa Cruz Biotechnology, Dallas, TX, USA) and 12 µg of “Bbs10 HDR Plasmid (h2)” (Santa Cruz Biotechnology, Dallas, TX, USA), using Lipofectamine 2000 (Thermo Fisher Scientific, Waltham, MA, USA) following the supplier instructions. The culture medium was replaced 48 h after the transfection with a selective medium containing 3 µg/mL puromycin (Santa Cruz Biotechnology, Dallas, TX, USA). The transfected cell pool (IMCD3-Bbs10^-/-^ pool) was kept in culture in a selective medium for several days with phosphate-buffered saline (PBS) (EuroClone, Paington, UK) and medium changes, to eliminate detached cells and select adherent puromycin-resistant cells. In the following weeks, the pool of resistant cells was properly diluted and plated to have separate colonies deriving from a single resistant cell clone. RFP signal is a surrogate marker of the efficiency of transfection. The colonies were then detached and kept in culture separately. IMCD3-Bbs10^-/-^) pool and four clones were tested to confirm the Bbs10 deletion, using qRT-PCR [32].

### 4.9. Generation of a Cell Line Stably Expressing BBS10 for Protein-Protein Interactions (PPIs)

Hek293t/17 wild type cells were seeded in a 10 cm diameter at the density of 1.5 × 103 cells/mm^2^ of the plate and kept in culture in a medium without antibiotics. After 24 h, cells were transfected with 10 µg of BBS10 cDNA ORF Clone, Human, N-DDK (Flag) tag expression plasmid (Sino Biological, SB) using Lipofectamine 2000 (Thermo Fisher Scientific, Waltham, MA, USA) following the supplier instructions. After 48 h from the transfection, the culture medium was replaced with a selective medium containing 75 µg/mL hygromycin (Santa Cruz Biotechnology, Dallas, TX, USA). The transfected cell pool (BBS10-Flag pool) was kept in culture in a selective medium for several days, with phosphate-buffered saline (PBS) (EuroClone, Paington, UK) washes and medium changes in order to eliminate detached cells and select adherent hygromycin-resistant cells. The colonies were then detached and kept in culture separately. The BBS10-flag pool and the clones were tested by WB to verify the presence of tagged protein expression.

### 4.10. Western Blot

Protein samples were fractionated by a 10% SDS-PAGE and transferred onto nitrocellulose membranes using a Trans-Blot Turbo Transfer System (Bio-Rad, Hercules, CA, USA). Membranes were blocked for 2 h at room temperature with 5% milk in PBS with a 0.2% Tween-20. Each primary antibody used for WB was incubated O/N at 4 °C in 5% milk in PBS with a 0.2% Tween-20. All antibodies used in this study are commercially available, from Sigma-Aldrich: TRIM65 (HPA021578) and Flag M2 (F1804); from Thermo Fisher Scientific: MID1 (PA538524); and from abcam: TAK1/MAP3K7 (ab109526). Immunoblot detections were carried out using horseradish peroxidase-conjugated antibodies (Claritry Western ECL Substrate, Biorad, Hercules, CA, USA) and enhanced chemiluminescence (Claritry Max Western ECL Substrate, Biorad, Hercules, CA, USA). Signals were acquired by Biorad Chemidoc. Densitometry analysis was performed by Image Lab^TM^ software.

### 4.11. Quantitative Real-Time PCR

For each qRT-PCR assay, 1.5 × 10^3^ cells/mm^2^ of mIMCD3 cells were seeded in a 6 cm diameter plate and kept in culture in standard conditions (see above). After 24 h, cells were washed twice with PBS and total RNA was extracted from primary keratinocytes or epidermis using EUROGOLD TriFast reagent (EuroClone, Paington, UK). In total, 500 ng of RNA were reverse transcribed using SuperScript™ VILO™ MasterMix (Thermo Fisher Scientific, Bremen, Germany). Then, qRT-PCR was carried out in a 7500 Real-Time PCR System PCR Thermal Cycler with appropriate primers using an SYBR^®^ Select Master Mix (Applied Biosystems, Monza, Italy). Gene expression levels of Bbs10, Pdk1, Hk-1, Ldha1, Pkm2, Acox1, Cpt1, Cpt2, and Glut1 were normalized to RNA polymerase II (POLR2A) and β2 microglobulin protein and calculated using the 2−∆∆Ct method. Average values from at least three independent experiments were graphically reported as relative units. Statistical significance was calculated by a two-tail unpaired t-test. The primer sequences are reported below (Table 5).

### 4.12. Mitotic Index and Colony Formation Assay

The miotic index indicated the percentage of cells undergoing mitosis or is defined as the ratio of the no. of cells in the dividing phase to the total number of cells observed. IMCD3-*Bbs10*^-/-^ cells and wild type cells were plated (1000 cells/well) in 6-well plates (Corning CoStar). For the colony formation assay, after 48 h, cells were washed and grown for four days until visible colonies were formed. Colonies were fixed with a 1:1 ratio of acetone:methanol and stained with 0.25% crystal violet (Sigma-Aldrich C3886-25 mg) in 25% methanol, washed, air-dried, and photographed. Crystal violet was dissolved in sodium citrate buffer (0.1 M (pH 4.2) and 50% ethanol) and absorbance was measured at λ = 490 nm. This assay was replicated three times. The statistical significance was examined by one-way ANOVA multiple comparisons (multiple comparison test).

### 4.13. MTT Assays

mIMCD3 Bbs10^-/-^ and wild type cells were seeded in a 96-well plate at a density of 0.5 ×104 cells/well and 1 × 104 cells/well in 100 μL of complete medium (DMEM F-12 D6421 SIGMA). A 3-(4,5-dimethyl thiazolyl-2)-2,5-diphenyltetrazolium bromide (MTT) solution (M2128-1G) was added at a final concentration of 0.05 mg/well and incubated for at 37 °C, and 5% CO2 for 4 hrs. The purple formazan crystals were dissolved by adding isopropanol with 0.04% HCl (1N). A spectrophotometer measured absorbance at λ = 570 nm (Perkin Elmer Version 1.09). The MTT assay was carried out after 24 h. MTT readings were recorded in triplicates, and the overall mean was plotted against the absorbance. The standard error of the mean (SEM) was calculated, and the statistical significance was examined by one-way ANOVA multiple comparisons (Dunnett’s multiple comparison test).

### 4.14. Intracellular ATP Measurement

ATP intracellular content was quantified in mIMCD3-Bbs10^-/-^ and wild type cells using an ATP Determination Kit (Thermo Fisher Scientific) for cellular extracts in which luciferase converts luciferin to oxyluciferin in the presence of ATP and oxygen. The assay is based on luciferase’s absolute requirement for ATP in producing light (560 nm, emission maximum at pH 7.8), according to ATP Determination Kit (Molecular Probe).

Preparation of the ATP standard curve: The ATP stock solution had a final concentration of 5 mM. The low ATP standard solutions were prepared by diluting the 5 mM stock solution in deionized water (dH_2_O). For this experiment, the ATP concentration usually ranged from 1 nM to 1 µM. The choice depended upon the sensitivity of the luminometer used. The reaction buffer contained water, 20X reaction buffer, dithiothreitol (DTT), D-luciferin, and the firefly luciferase enzyme. For the ATP assay, cells were lysed in ice with a specific lysis buffer (Tris-HCl ph8 50 mM, NaCl 150 mM, 0.5% NP-40, EDTA ph8 2 mM, and 1X phosphatase inhibitors) and were placed on wet ice for 20 min. After, 10 µL of the cell’s lysate was mixed with 90 µL of the reaction buffer in a 96-well black plate, and ATP was quantified by using a Perkin Elmer Enspire plate reader (Perkin Elmer, Waltham, MA, USA). The emitted luminescence signal is directly proportional to the ATP content of cells. The ATP intracellular content was normalized to the number of cells. The ATP measurement was performed three times after cell seeding, at 24, 48, and 72 h, respectively. The Oligomycin, a selective inhibitor of the ATPase proton channel, was used at a final concentration of 30 µg/mL for 5 h.

### 4.15. Immunoprecipitation

Lysis Buffer: 1X TBS (250 mM Tris-HCl pH 7.4, 1.40 M NaCl, 0.03 M KCl), 1 mM EDTA, 1% Triton X-100, 10% glycerol, and proteinase inhibitors. Washing Buffer: 1X TBS (250 mM Tris-HCl pH 7.4, 1.40 M NaCl, 0.03 M KCl), 1 mM EDTA, 10% glycerol, and proteinase inhibitors. The selected cell lines expressing the BBS10-flag were grown in an FBS-free medium in starvation for 48 h. Then, HEK293 cells expressing the BBS10-flag and the GFP-flag vector used as control were lysates with the Lysis Buffer.

Total protein extracts were pre-cleared with mouse IgG agarose beads and incubated ON at 4 °C with M2 anti-FLAG agarose-conjugated antibody beads (Sigma). The control experiment was obtained by immunoprecipitation of GFP-flag vector-transfected cells with anti-FLAG agarose beads to rule out unspecific retained proteins as described. Non-retained proteins were then incubated with M2 anti-FLAG agarose-conjugated antibody beads (Sigma) overnight at 4 °C. Beads were washed with washing buffer. Retained protein complexes were eluted with 3XFLAG peptide and subjected to precipitation with methanol/chloroform. The protein mixture was vacuum-dried and re-suspended in 10% SDS buffer to perform in situ protein hydrolysis with a S-TrapTM micro spin column for interactors identification.

### 4.16. Interactome Analysis

The immunoprecipitated sample after resuspension in 10% SDS buffer was reduced with the addition of TCEP (Sigma-Aldrich, St. Louis, MO, USA) to a final concentration of 100 mM and alkylated with the addition of iodoacetamide (Sigma-Aldrich, St. Louis, MO, USA) to a final concentration of 50 mM. Aqueous phosphoric acid was added to a final concentration of 1.2%. Colloidal protein particulate was formed with the addition of six times the sample volume of an S-Trap binding buffer (90% aqueous methanol, 100 mM TEAB, pH 7.1). The mixtures were put on the S-Trap micro columns and centrifuged at 4000× *g* for 30 s. The columns were washed three times with 150 µL S-Trap binding buffer and centrifuged at 4000× *g* for 30 s between washes. The mixtures were digested using 3 µg of trypsin (Promega) at 47 °C for 1 h. After elution, peptides were vacuum dried and resuspended in 35 µL of 10% ACN and 0.1% TFA in HPLC-grade water prior to MS analysis [33,34].

### 4.17. NanoLC-MS/MS Measurements

Four independent biological replicates for the BBS10-flag and four replicates for the GFP-flag were analyzed by nLC-MS/MS. Samples were resuspended in 100 µL of 10% ACN and 0.1% TFA in HPLC-grade water. For each run, 5 µL were injected in a nanoRSLC-Q Exactive PLUS (RSLC Ultimate 3000) (Thermo Scientific, Waltham MA, USA). Peptides were loaded onto a µ-precolumn (Acclaim PepMap 100 C18, cartridge, 300 µm i.d.×5 mm, 5 µm) (Thermo Scientific) and were separated on a 50 cm reversed-phase liquid chromatographic column (0.075 mm ID, Acclaim PepMap 100, C18, 2 µm) (Thermo Scientific). Chromatography solvents were (A) 0.1% formic acid in water and (B) 80% acetonitrile and 0.08% formic acid. Peptides were eluted from the column with the following gradient 5% to 40% B (120 min), 40% to 80% (1 min). At 121 min, the gradient stayed at 80% for 5 min and, at 127 min, it returned to 5% to re-equilibrate the column for 20 min before the next injection. Two blanks were run between each series to prevent sample carryover. Peptides eluting from the column were analyzed by data-dependent MS/MS using the top-10 acquisition method. Peptides were fragmented using higher-energy collisional dissociation (HCD). Briefly, the instrument settings were as follows: the resolution was set to 70,000 for MS scans and 17,500 for the data-dependent MS/MS scans in order to increase speed. The MS AGC target was set to 3.10^6^ counts with a maximum injection time set of 200 ms, while the MS/MS AGC target was set to 1.10^5^ with a maximum injection time set to 120 ms. The MS scan range was from 400 to 2000 m/z [32].

### 4.18. MS Raw-Files Data Processing

The MS files were processed with the MaxQuant software version 1.6.5.0 and searched with the Andromeda search engine against the database of Homo sapiens from Swiss-Prot 07/2017. To search parent mass and fragment ions, we set an initial mass deviation of 4.5 ppm and 20 ppm, respectively. The minimum peptide length was set to eight amino acids and strict specificity for trypsin cleavage was required, allowing up to two missed cleavage sites. Carbamidomethylation (Cys) was set as a fixed modification; oxidation (Met) and N-term acetylation were set as variable modifications. Match between runs was not allowed. The false discovery rates (FDRs) at the protein and peptide levels were set to 1%. The reverse and common contaminants hits were removed from MaxQuant output. Proteins identified in all biological replicates of the BBS10-flag and GFP-flag interactomes were enrolled in the proteome dataset and processed in the subsequent quantitative analysis. The proteins were quantified according to the MaxQuant label-free algorithm using MS1 peak intensity as quantitative parameters.

The datasets of BBS10-flag and GFP-flag interactomes were analyzed with the Perseus software version 1.6.0.7 (www.perseusframework.org, MPI of Biochemistry, Martinsried, Germany; accessed on 13 April 2022). Specifically, MS1 peak intensity data were log2-transformed. Missing values were replaced by creating a Gaussian distribution of random numbers with a standard deviation of 30% relative to the standard deviation of the measured values and three standard deviation downshifts of the mean. The log2 protein difference of the intensities was then calculated between the two analyzed groups in order to describe the variation in protein abundance.

After, the list of interactors was subjected to clusterization using STRING (Search Tool for the Retrieval of Interacting Genes) online version 11 (STRING CONSORTIUM 2020) [34], in order to generate the interacting network. The identified networks were evaluated by a significant interaction score as a negative logarithm of the *p*-value.

## Figures and Tables

**Table 1:** Main features of BBS patients and controls: the pilot study.

Features	BBS Patients (*n* = 14)	Controls (*n* = 20)	*p*
Age (years)	28.8 ± 6.6	32.2 ± 5.5	0.11
Gender (M)	7/14	10/20	-
eGFR (ml/min/1.73 m^2^)	99.4 ± 24.5	101.3 ± 11.23	0.08
BMI (kg/m^2^)	30.8 ± 6.5	27.59 ± 5.5	0.08
SBP (mmHg)	111.3 ± 11.9	115.8 ± 9.4	0.2
DBP (mmHg)	80.1 ± 7.3	77.0 ± 4.4	0.2
Retinal dystrophy	Present in 13 out of 14	-	
Learning disabilities	Present in 9 out of 14	-	
Polydactyly	Present in 10 out of 14	-	

Data are presented as mean ± SEM. The statistical significance of the difference in metabolite concentrations between conditions (BBS and controls) was evaluated by an unpaired parametric t-test with Welch correction or unpaired no parametric Mann–Whitney test when data failed the D’Agostino normality test.

**Table 2:** Urine concentrations (μM) of metabolites differentially represented in BBS patients vs. controls in the pilot study.

Features	BBS Patients (*n* = 14)	Controls (*n* = 20)	*p*
Lactic acid	18.41 ± 2.22	10.29 ± 0.49	0.000198
β-lactic acid	1.68 ± 0.39	0.95 ± 0.09	0.037841
Pyruvic acid	8.50 ± 0.75	6.02 ± 0.43	0.004451
3-Hydroxyisobutyric acid	6.99 ± 0.79	4.16 ± 0.40	0.001547
2-Ethyl-3-hydroxypropionic acid	3.35 ± 0.57	1.44 ± 0.25	0.001746
Ethylmalonic acid	4.70 ± 0.50	2.99 ± 0.39	0.009981
Succinic acid	15.31 ± 0.90	11.90 ± 0.50	0.001210
Fumaric acid	0.80 ± 0.07	0.58 ± 0.04	0.006470
Erythro-pentonic acid	1.93 ± 0.08	1.55 ± 0.07	0.001386
Erythronic acid	1.16 ± 0.08	0.93 ± 0.06	0.027748
2 Hydroxyglutaric acid	1.91 ± 0.27	1.29 ± 0.10	0.022481
4 Hydroxyphenilacetic acid	8.79 ± 1.79	5.33 ± 0.93	
Quinolinic acid	0.51 ± 0.08	0.30 ± 0.02	0.004988
Retinoic acid	2.12 ± 0.25	1.45 ± 0.10	0.010155
4 Hydroxyphenilactic acid	1.20 ± 0.20	0.79 ± 0.15	
Palmitic acid	40.15 ± 3.53	55.33 ± 3.81	0.008682
Palmitelaidic acid	1.54 ± 0.21	2.26 ± 0.17	0.010924
Oleic acid	19.91 ± 2.08	28.67 ± 2.17	0.008521
(E)-trans 9 Octadecenoic acid	5.51 ± 0.79	9.34 ± 0.87	0.003948
Stearic acid	45.68 ± 4.37	70.00 ± 4.96	0.001448

Data are presented as mean ± SEM. The statistical significance of the difference in metabolite concentrations between conditions (BBS and controls) was evaluated by an unpaired parametric t-test with Welch correction or an unpaired no parametric Mann–Whitney test when data failed the D’Agostino normality test.

**Table 3:** Plasma and urinary markers of proximal tubule (PT) function.

Features	BBS Patients (*n* = 14)	Controls (*n* = 20)	*p*
Plasma			
Na^+^ (mEq/L)	140.5 ± 1.9 ^1^	140.6 ± 1.7	0.86
K^+^ (mEq/L)	4.1 ± 0.2	4.6 ± 0.3	0.8
Phosphate (mg/dL)	3.7 ± 0.6	3.9 ± 0.3	0.42
Uric acid (mg/dL)	5.5 ± 1.5	3.9 ± 0.9	0.25
Urine			
Ca^++^/creatinine ratio	0.08 ± 0.04	0.09 ± 0.04	0.6
Glycosuria	neg	neg	
FeUA(%)	5.6 ± 4.13	6.0 ± 1.3	0.3
FePO_4_(%)	11.9 ± 4.4	12.1 ± 6.6	0.9
TmP/GFR	1.14 ± 0.3	1.10 ± 0.3	0.8
Amino-acids			
Taurine	77.7 ± 46	65.6 ± 39	0.44
Aspartic acid	4.08 ± 8.8	0.54 ± 39	0.09
Threonine	15.5 ± 8.6	17.04 ± 10.6	0.69
Serine	37.75 ± 19	36.05 ± 17.4	0.81
Asparagine	17.9 ± 14.8	15.9 ± 8.6	0.63
Glutamic acid	0.17 ± 0.55	1.19 ± 0.85	0.001 *
Glutamine	38.25 ± 13.3	42.7 ± 20.4	0.53
Proline	4.6 ± 15.5	0.54 ± 2.4	0.27
Glycine	112 ± 123.01	118 ± 55.5	0.84
Alanine	38.4 ± 37.4	25.3 ± 15.1	0.19
Valine	3.33 ± 1.8	3.49 ± 1.6	0.80
Cystine	12.8 ± 19.1	7.70	0.28
Methionine	4.58 ± 2.2	5.45 ± 2.6	0.34
Leucine	7.08 ± 5.4	6.11 ± 6.5	0.12
Tyrosine	17.58 ± 8.5	12.93 ± 7.2	0.11
Phenylalanine	9.83 ± 3.7	7.58 ± 2.7	0.11
Ornithine	2.92 ± 6.4	1.75 ± 2.07	0.47
Citrulline	1.5 ± 2.4	Undetectable	
Lysine	66.50 ± 80.0	44.08 ± 36.7	0.28
Histidine	91.33 ± 74.6	65.04 ± 20.1	0.30
Tryptophan	undetectable	undetectable	
Arginine	1.58 ± 4.3	0.9 ± 1.13	0.44

Data are presented as mean ± SEM. The statistical significance of the difference in metabolite concentrations between conditions (BBS and controls) was evaluated by an unpaired parametric t-test with Welch correction or unpaired no parametric Mann–Whitney test when data failed the D’Agostino normality test. * These parameters (with underlines) did not differ between patients and controls, with the exception of glutamic acid, suggesting no overt PT dysfunction in basal conditions.

**Table 4:** Major features of BBS patients and controls: *The Confirmation study*.

Features	BBS_noCKD eGFR > 90 mL/min/1.73 m^2^ (*n* = 18)	CTR_HV (Healthy Volunteers) (*n* = 18)	*p* (BBS no_CKD vs. ctr_hv)	BBS_CKD eGFR > 90 mL/min/1.73 m^2^ (*n* = 13)	CTR_CKD (Healthy Volunteers) (*n* = 13)	*p* (BBS no_CKD vs. ctr_hv)
Age (mean ± SD)	26.05 ± 7.1	30 ± 7	ns	32.92 ± 9.8	34.21 ± 12	ns
Gender (F)	12/18	8/17		5/13	8/13	
eGFR (ml/min/1.73 m^2^)	116.6 ± 13	98.4 ± 5.7	ns	49.9 ± 24	39 ± 22	ns
BMI (kg/m^2^)	30.5 ± 5.3	27.5 ± 3.7	ns	35.8 ± 9.3	32.7 ± 5.4	ns
Retinal dystrophy	18/18	-		13/13	-	

Data are presented as mean ± SEM. The statistical significance of the difference in metabolite concentrations between conditions (BBS-noCKD vs. CTR_HV and BBS_CKD vs. CTR_CKD) was evaluated by an unpaired parametric t-test with Welch correction (ns = not significant) or unpaired no parametric Mann–Whitney test (ns = not significant) when data failed the D’Agostino normality test.

**Table 5:** The primer sequences.

β2 Microglobin	GGTCTTTCTGGTGCTTGTTCT	TATGTTCGGCTTCCCATTCTC
Polr2a	GGATGAATTGAAGCGGATGTC	CACTCGGTCATGTTTCCTGC
Bbs10	CAAGTGTTGTGTACGAAGCC	CACACGCCACTATCATCCTG
Ldha1	GTGGAGTGGTGTGAATGTTG	TCACCTCGTAGGCACTGTCC
Hk-1	GCCGCCATTGAAACGGATAAG	TGCTGGACCGATACGCAGTC
Pdk1	GGCGGCTTTGTGATTTGTAT	ACCTGAATCGGGGGATAAAC
Pkm2	TGTCTGGAGAAACAGCCAAG	TCCTCGAATAGCTGCAAGTG
Glut-1	GTCGGCCTCTTTGTTAATCG	CACATACATGGGCACAAAGC
Cpt1	GGTCTTCTCGGGTCGAAAGC	TCCTCCCACCAGTCACTCAC
Cpt2	CAATGAGGAAACCCTGAGGA	GATCCTTCATCGGGAAGTCA
Acox1	CTTGGATGGTAGTCCGGAGA	TGGCTTCGAGTGAGGAAGTT

## Data Availability

Original data will be provided by authors if required.

## Supplementary Materials

The following supporting information can be downloaded at: https://www.mdpi.com/article/10.3390/ijms23169420/s1.
