# Supplementary material for: Multi-Omics Studies Unveil Extraciliary Functions of BBS10 and Show Metabolic Aberrations Underlying Renal Disease in Bardet–Biedl Syndrome
Source: Int J Mol Sci. 2022 Aug 20;23(16):9420. doi: 10.3390/ijms23169420 (PMC9409368; doi:10.3390/ijms23169420)
Supplement: Supplementary file 1 [file ijms-23-09420-s001.zip › Supplemental Figure S1.pdf]

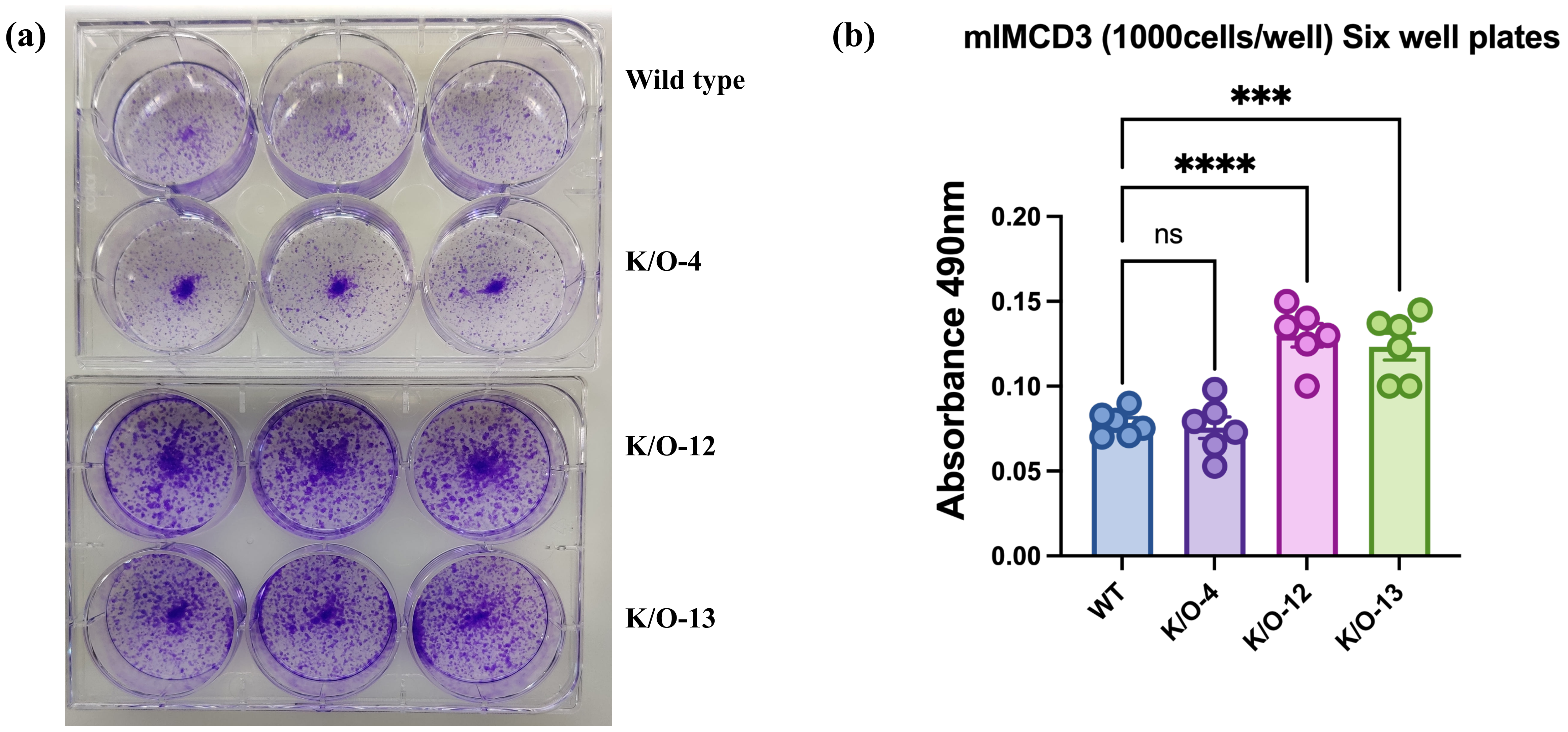

Supplementary Figure S1: In-vitro cell viability by clonogenicity assay. (a) Colonies of wild type cells and three different IMCD3-Bbs10-/- cellular clones were stained by using crystal violet. (b) Absolute quantification of cell survival was performed by measuring the absorbance at λ = 490 nm. The assay was performed using six well plates and including three different IMCD3-Bbs10 −/− clones. The statistical significance of the difference between three different IMCD3-Bbs10-/- cellular clones (K/O-4, K/O-12, K/O-13) and controls (WT) were evaluated by ordinary one-way ANOVA test and Hold-Sidak's multiple comparison test (*p<0.05, **p<0.01, ***p<0.001 ****p<0.0001, ns = not significant).
